# How Seasonality and Control Measures Jointly Determine the Multistage Waves of the COVID-19 Epidemic: A Modelling Study and Implications

**DOI:** 10.3390/ijerph19116404

**Published:** 2022-05-25

**Authors:** Yangcheng Zheng, Yunpeng Wang

**Affiliations:** 1 State Key Laboratory of Organic Geochemistry, Guangzhou Institute of Geochemistry, Chinese Academy of Sciences, Guangzhou 510640, China; zhengyangcheng@gig.ac.cn; 2 CAS Center for Excellence in Deep Earth Science, Guangzhou 510640, China; 3 University of Chinese Academy of Sciences, Beijing 100049, China

**Keywords:** COVID-19, multi-stage, control measures, seasonality, SEIR model

## Abstract

The current novel Coronavirus Disease 2019 (COVID-19) is a multistage epidemic consisting of multiple rounds of alternating outbreak and containment periods that cannot be modeled with a conventional single-stage Suspected-Exposed-Infectious-Removed (SEIR) model. Seasonality and control measures could be the two most important driving factors of the multistage epidemic. Our goal is to formulate and incorporate the influences of seasonality and control measures into an epidemic model and interpret how these two factors interact to shape the multistage epidemic curves. New confirmed cases will be collected daily from seven Northern Hemisphere countries and five Southern Hemisphere countries from March 2020 to March 2021 to fit and validate the modified model. Results show that COVID-19 is a seasonal epidemic and that epidemic curves can be clearly distinguished in the two hemispheres. Different levels of control measures between different countries during different seasonal periods have different influences on epidemic transmission. Seasonality alone cannot cause the baseline reproduction number *R*_0_ to fall below one and control measures must be taken. A superposition of a high level of seasonality and a low level of control measures can lead to a dramatically rapid increase in reported cases.

## 1. Introduction

The current novel Coronavirus Disease 2019 (COVID-19) is a multistage epidemic consisting of multiple rounds of alternating outbreak and containment phases. In a conventional single-stage epidemic, the number of daily new confirmed cases increases over time, gradually decreases after reaching its maximum, and eventually declines to zero despite sporadic relapses [1,2]. However, in a multistage epidemic, the number increases, reaches the maximum, and declines but does not return to zero. Instead, a new round of outbreak occurs, repeating the above steps [3]. By 20 March 2022, 470,1098,898 confirmed cases and 6,098,267 deaths have been reported, and the number continues to rise steadily [4]. So far, most countries have experienced multiple rounds of large-scale outbreaks, but the driving factors of the multistage epidemic and its specific influences remain to be elucidated.

Control measures may be an important factor of the multistage epidemic [5,6], which interrupt transmission chains by reducing contact between people [7], and different levels of control measures may have a different impact on the transmission of COVID-19 [8,9,10]. Mild control measures include wearing masks, maintaining social distance, and routine temperature measurements, while strict control measures include closing public facilities (e.g., schools and restaurants), border closures, and traffic restrictions [10]. In China’s most severe case during the first round of the outbreak in 2020, the entire country was locked down and people were told to stay home unless they went out for daily needs, which effectively prevented the transmission of COVID-19 [11,12,13]. In the case of the United States, control measures could not be fully implemented during both the outbreak and containment phases, resulting in a significantly higher number of new cases confirmed daily compared with other countries. Therefore, the formulation and evaluation of the extent and impact of control measures are important for the interpretation of the multistage patterns.

Despite the control measures, seasonality could be another important factor influencing the multistage epidemic. It is still controversial whether the transmission of COVID-19 follows the similar seasonal patterns as other seasonal epidemics [14,15,16,17]. Epidemic curves show that outbreaks are concentrated in two hemispheres in different time periods [4]. In Northern Hemisphere countries, the number of new cases confirmed dramatic daily increases between November and January, whereas in Southern Hemisphere countries, a distinct outbreak period is observed between May and August. A number of studies have attempted to establish the relationship between the occurrence of COVID-19 and seasonally varying meteorological factors. Confirmed cases were found to be concentrated in air temperatures of 5 to 15 °C and absolute humidity of 3 to 10 g/m^3^ [18]. Experimental data and regional analyses show that coronavirus has a lower survival rate in a high temperature environment, suggesting that the virus may be less infectious in summer [19]. Lower wind speed significantly correlates with higher COVID-19 cases by affecting the transmission of aerosols [20,21], which are considered important virus carriers [22]. Despite the evidence for seasonality, there is debate that COVID-19 is not seasonal. Some studies find no relationship between temperature and the occurrence of COVID-19 [23,24,25] or state that the relationship exists only at the global level, but not at the regional level [26]. Given the recent outbreak in the winter of 2022, there is growing evidence that COVID-19 is a seasonal epidemic, but for policy decisions, the influences of seasonality have yet to be formulated and verified in model simulations. 

Many studies have been conducted to examine the influences of control measures or seasonality separately, but few studies have been conducted to examine the joint influences of these two factors. In addition, most modeling studies for COVID-19 epidemic simulation focus on the first round of the outbreak and are not able to simulate the multistage epidemic. In this paper, we attempt to formulate the influences of seasonality and control measures on the transmission of the COVID-19 epidemic. We modify the most commonly used Suspected-Exposed-Infectious-Removed (SEIR) epidemic model by incorporating the influences of these two factors. We simulate the epidemic curves of twelve countries from two hemispheres from March 2020 to March 2021 using our modified model and interpret how these two factors interact and ultimately influence the transmission of the COVID-19 epidemic.

## 2. Materials and Methods

Seven Northern Hemisphere countries (United States, United Kingdom, Italy, France, Germany, Spain, and Turkey) and five Southern Hemisphere countries (Brazil, Chile, Australia, South Africa, Argentina) were selected and daily new confirmed cases from these countries are collected daily [4]. The selected countries, where considerable confirmed cases have been reported and where obvious multistage patterns are observed, are representative cases for the study of seasonality between two hemispheres. Countries near the equator, whose climates do not vary as much with the seasons, are not included [27]. The study period for each country begins when the number of new daily confirmed cases exceeds 100, to avoid the high uncertainty in reported data due to limited testing capabilities in the very early phase of the epidemic, and includes a full year for an entire seasonal cycle (approximately from March 2020 to March 2021). The influences of new variants and vaccination are excluded from the study period, and only seasonality and control measures are formulated. 

The exact timing of implementation and mitigation of each round of control measures are gathered from official news reports or announcements (Table 1) to formulate the influences of control measures. An outbreak period is defined as the period from the request for control measures to the mitigation of control measures, while a containment period is inversely defined as the period from the mitigation of control measures to the request for a new round of control measures. In the selected countries, at least two rounds of outbreaks and weakening are observed.

The SEIR model divides the population into four subpopulations, the suspect population (*S*), the exposed population (*E*), the infectious population (*I*), and removed population (*R*), whose relationship can be formulated by the following differential equations:

(1) $$\frac{dS\left(t\right)}{dt}=-\frac{\gamma R_{0}\left(t\right)}{N}S\left(t\right)I\left(t\right),$$

(2) $$\frac{dE\left(t\right)}{dt}=\frac{\gamma R_{0}\left(t\right)}{N}S\left(t\right)I\left(t\right)-\alpha E\left(t\right),$$

(3) $$\frac{dI\left(t\right)}{dt}=\alpha E\left(t\right)-\gamma I\left(t\right),$$

where *R*_0_ is the baseline reproduction number (defined as the average number of secondary infections caused by a single infected individual in a fully susceptible population [28]), *N* is the total population, *γ* is the removal rate (fixed at 0.34), and *α* is the infection rate (fixed at 0.19). The model is fitted with daily data of new confirmed cases and therefore the equation of the removed population is not presented here. 

In a conventional single-stage SEIR model, *R*_0_ is usually fixed as a fixed value. However, in our multistage model, we replace *R*_0_ in Equations (1) and (2) with an adjusted, time-varying $R_{0}^{\prime }\left(t\right)$, which is defined as follows:

(4) $$R_{0}^{\prime }\left(t\right)=R_{0}\left(t\right)q\left(t\right),$$

where *t* denotes the days since the study period, *q*(*t*) is a time-varying quarantine index, and *R*_0_(*t*) is a time-varying and periodic parameter defined by the following periodic function to formulate the seasonality of COVID-19:

(5) $$R_{0}\left(t\right)=A_{0}cos\;\left(\frac{2\pi }{365}t+\omega \right)+A_{1},$$

with *A*_0_ being the amplitude of seasonal fluctuation, *A*_1_ the constant term, *ω* the initial phase, and the length of the periodic cycle is set as 365 days.

We construct a time-varying quarantine index q(t), which falls between (0, 1], to formulate the influences of control measures. The quarantine index is a parameter to give a rough but quantitative estimation about the strict levels of control measures implemented in the whole country. In other words, to what extend can $R_{0}\left(t\right)$ be brought down by control measures. In a mathematical sense, when q(t) is equal to zero, $R_{0}^{\prime }\left(t\right)$ is zero and the epidemic is cut off completely. When q(t) is equal to one, $R_{0}^{\prime }\left(t\right)$ is equal to $R_{0}\left(t\right)$ and the epidemic spreads without any restrictions. In reality, q(t) cannot be one except the period without any control measures in the very early epidemic stage. In addition, q(t) cannot be zero because so far the transmission of COVID-19 cannot be cut off completely in any country. Thus, a lower bound $q_{o}$ and an upper bound $q_{m}$, with 0 *<*$\;q_{o}\;$*<*$\;q_{m\;}$*≤* 1, are set to constrain the values of q(t), and the variations of q(t) during outbreak and containment periods are shown in Equations (6) and (7):

(6) $$\mathrm{outbreak}\;\mathrm{period}\;q\left(t\right)=\left(q_{m}-q_{o}\right)e^{\left(\varepsilon_{o}\left(t-t_{o}\right)\right)}+q_{o},$$

(7) $$\mathrm{containment}\;\mathrm{period}\;q\left(t\right)=\left(q_{o}-q_{m}\right)e^{\left(\varepsilon_{m}\left(t-t_{m}\right)\right)}+q_{m},$$

with subscripts *o* and *m* denoting the outbreak period and containment period, respectively, *t_o_* and *t_m_* being the start dates of outbreak period and containment period. Parameters $\varepsilon_{o}$ and $\varepsilon_{m}$ are applied to controlling the the ascending and descending speed of exponent functions in Equations (6) and (7). In other words, higher values of $\varepsilon_{o}$ mean that the epidemic curves climb rapidly during outbreak periods, while lower values of $\varepsilon_{m}$ mean that the epidemic curves decline slowly during containment periods. For brevity, the values of $q_{o}$, $q_{m}$, $\varepsilon_{o}$ and $\varepsilon_{m}$ are assumed to share the same value in each epidemic stage. It should be noted that $q_{m}$ is set as one in the earliest stage as there were no control measures. In the modified SEIR model, in total seven parameters ($q_{o}$, $q_{m}$, ω, $A_{0}$, $A_{1}$, $\varepsilon_{o}$, and $\varepsilon_{m}$) should be fitted. Genetic algorithm is applied in this study to approximating the best parameters combination [29]. To accelerate the solution, coarse lower and upper bounds of these seven parameters are explored upfront, as shown in Table 2.

## 3. Results

**Figure 1:**
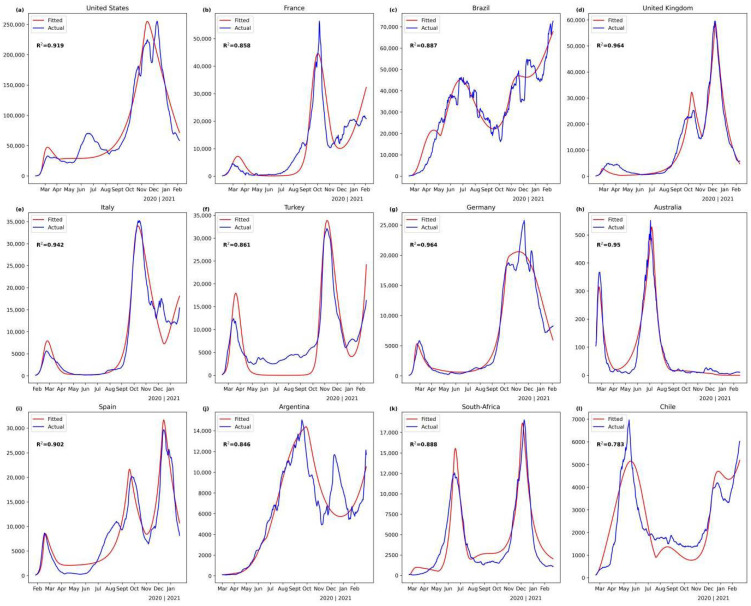
The curves of fitted and actual daily new confirmed cases for the selected countries. (**a**) United States; (**b**) France; (**c**) Brazil; (**d**) United Kingdom; (**e**) Italy; (**f**) Turkey; (**g**) Germany; (**h**) Australia; (**i**) Spain; (**j**) Argentina; (**k**) South-Africa; (**l**) Chile.

Our modified SEIR model performs well in fitting the multistage COVID-19 epidemic curves with *R*^2^ ranging from 0.781 to 0.969 (Figure 1). The best fitting performance, with *R*^2^ greater than 0.94, is obtained in Germany, the United Kingdom, Italy, and Australia, where two or three rounds of alternating outbreak and downturn periods can be simulated. Our model manages to capture the main trend of the epidemic curves during each period and neglect the smaller noise (e.g., Brazil). In some cases, despite high fitness, some outbreaks cannot be captured. For example, the second round of the outbreak period in the United States and Argentina is ignored.

**Figure 2:**
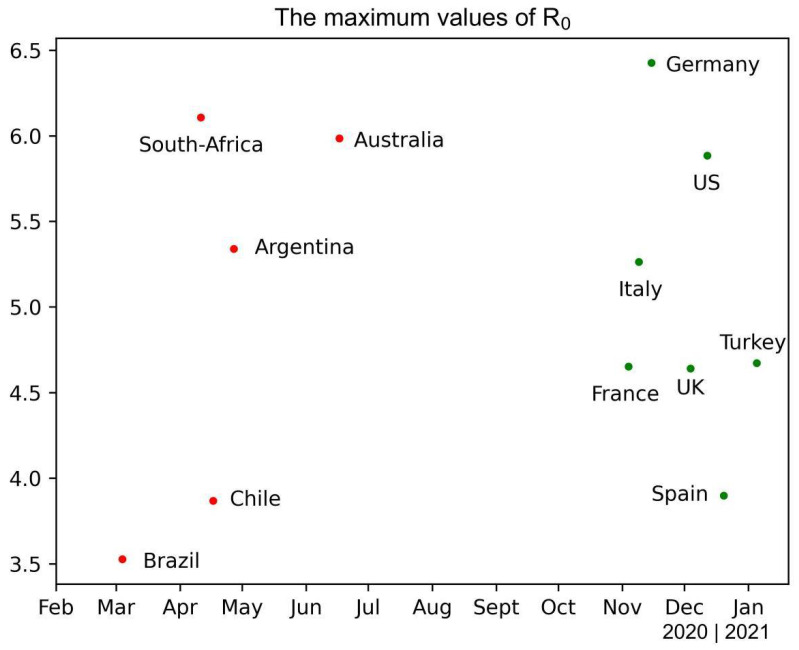
The maximum values of $R_{0}$ of the selected countries.

The results of the seven parameters with best fitting performance are listed in Table 3. According to the definition of the periodic function (Equation (5)), $R_{0}\left(t\right)$ reaches the maximum value at $A_{0}\;$+$\;A_{1}$ (*t* = 365-ω) and the minimum value at $A_{1}$−$A_{0}$ (*t* = 182-ω). The maximum value ranges from 3.53 (Brazil) to 6.42 (Germany) while the minimum value ranges from 1.02 (Turkey) to 4.76 (United States). The lowest minimum value of 1.02 in Turkey indicates that $R_{0}\left(t\right)$ cannot fall below one with only seasonal fluctuation and control measures must be implemented. The maximum values of seasonally fluctuating $R_{0}\left(t\right)$ of selected countries are projected in Figure 2. Countries from two hemispheres can be clearly distinguished, with $R_{0}\left(t\right)$ reaching maximum values in November and January in the Northern Hemisphere and March and June in the Southern Hemisphere. The value of $q_{o}$ ranges from 0.09 (Australia) to 0.32 (Brazil), indicating that control measures can reduce the capability to transmit during the outbreak period to at least one-third. The value of $q_{m}$ falls between 0.21 (United States) and 0.79 (Turkey), implying that the transmission capability can increase to at most four-fifths during the containment period. The major turning points of the epidemic curves are near the initial dates of the outbreak or containment periods when control measures are implemented or contained. In most countries, implementation of control measures can result in a significant decrease in new daily confirmed cases.

**Figure 3:**
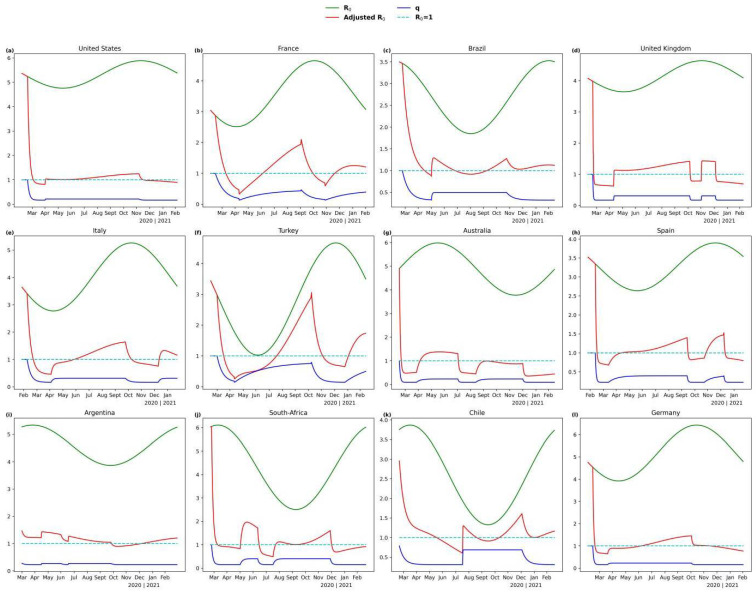
The curves of $R_{0}\left(t\right)$, $R_{0}^{\prime }\left(t\right)$ and *q(t)* of each selected country. (**a**) United States; (**b**) France; (**c**) Brazil; (**d**) United Kingdom; (**e**) Italy; (**f**) Turkey; (**g**) Germany; (**h**) Australia; (**i**) Spain; (**j**) Argentina; (**k**) South-Africa; (**l**) Chile.

The relations of $R_{0}\left(t\right)$, $R_{0}^{\prime }\left(t\right)$, and *q(t)* are shown in Figure 3. At the earliest stage of the epidemic, when no control measures are implemented and COVID-19 spreads unimpeded, *q*(t) is equal to one and $R_{0}^{\prime }\left(t\right)$ is equal to $R_{0}\left(t\right)$. During the outbreak periods, control measures are implemented and *q*(t) falls exponentially from maximum to minimum (from $q_{m}$ to $q_{o}$) while $R_{0}^{\prime }\left(t\right)$ also decreases, and the outbreak is prevented. An outbreak period is followed by a containment period, in which control measures are mitigated, with *q* resuming to $q_{m}$ and $R_{0}^{\prime }\left(t\right)$ rebounding as well. Outbreak and containment periods increase alternately, with *q*(t) varying between $q_{m}$ and $q_{o}$ and $R_{0}^{\prime }\left(t\right)$ goes up and down around one.

## 4. Discussion

The dates when the periodic $R_{0}\left(t\right)$ reach maximum values in two hemispheres are concentrated in two distinct periods, demonstrating that the transmission ability of COVID-19 indeed fluctuates seasonally. The amplitudes of $R_{0}\left(t\right)$ vary among countries, which may be attributed to the atmospheric pollution [30,31], demographic structure [20,32], healthcare condition, and other environmental or social factors [33]. In general, our model performs better in the northern hemisphere cases than the southern hemisphere cases. In some southern hemisphere cases (i.e., Argentina, Chile), the anti-seasonal outbreaks in January, 2021 could not be explained by seasonality and further efforts should be put on. 

The formulation of the influences of control measures is significant for explaining the different forms of epidemic curves among selected countries. We define a time-varying quarantine index *q*(t) and assume that q rises or falls in an exponential form after the implementation or mitigation of control measures, and stays stable when it reaches maximum or minimum values. Our early attempts show that an exponential function has better performance in formulating the variation processes of quarantine index compared with a polynomial function or sinusoidal function. The upper bound $q_{m}$ is assumed to be greater than the lower bound $q_{o}$ in simulation, representing a lower level of control measures in the containment period than that of the outbreak period. The values $\varepsilon_{o}$ and $\varepsilon_{m}$ control the speed at which the quarantine index reaches its maximum or minimum values, in other words, how fast the restriction or mitigation policies take effect. In practice, the upper bound of $\varepsilon_{o}$ and $\varepsilon_{m}$ is set to three, which means that q reaches its maximum or minimum values within one day; thus, a value greater than three is meaningless. The quarantine index relies on the exact dates when the policies are claimed, and it does not perform well if these dates cannot be collected. For instance, control measures in the United States are claimed state-to-state rather than nationwide, and thus the second round of outbreak is not caught by this model. In addition, the quarantine index may be greater than one in some extreme cases with the contact rate of the populations far beyond the usual (i.e., the large-scale concerts or sports events), which are not considered in this study.

**Figure 4:**
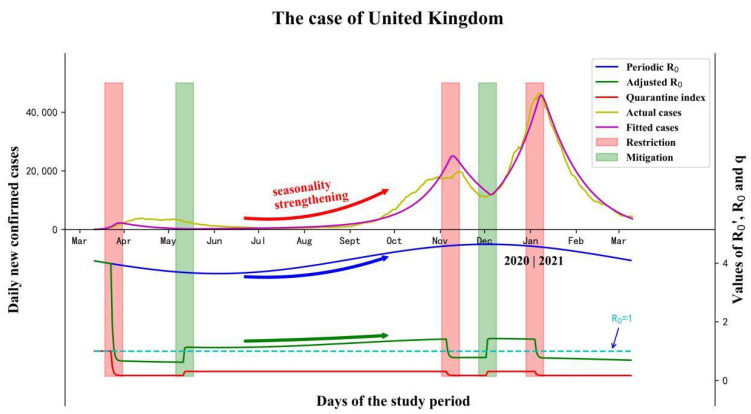
The co-influences of seasonality and control measures on the multi-stage COVID-19 epidemic in the case of the United Kingdom.

Seasonality and control measures are the two most significant driving factors that influence the transmission ability of COVID-19, and a single factor itself is not able to interpret the different multi-stage epidemic curves. The epidemic curve of the United Kingdom is an ideal case to reveal the combined effects of these two factors (Figure 4). At the earliest stage, the epidemic spread without any restrictions and $R_{0}^{\prime }\left(t\right)$ was equal to $R_{0}\left(t\right)$. The first round of control measures was implemented on 23 March 2020, after which *q*(t) dropped from one to $q_{o}$ and $R_{0}^{\prime }\left(t\right)$ dropped as well. The control measures were mitigated on 11 May 2020, and the daily confirmed cases maintained a low level during May and August. With the seasonality force strengthening, the curve started to rise up in September, 2020 and the second round of outbreak occurred in October, 2020. With control measures being tightened again on 5 November 2020, $R_{0}^{\prime }\left(t\right)$ fell down to less than one and a short-term decline was recorded. However, when the cases were still at a high level, control measures were mitigated immediately on 2 December 2020. Under the superposition of high values of *q*(t) and $R_{0}\left(t\right)$ during November and January, the cases rebound rapidly and soon the third round of outbreak occurred. It’s an interesting phenomenon that the first round of mitigation resulted in a period with cases at a low level, while the second round of mitigation resulted in a rapid rebound, and this phenomenon can be observed in most northern hemisphere countries (i.e., United Kingdom; France; Italy; Spain). The major difference between these two rounds of mitigation demonstrates the existence of seasonality. 

It is well known that the epidemic enters an expansion stage when the initial baseline reproduction number *R*_0_ is greater than one, and that a large-scale outbreak may subsequently occur. However, few studies have provided a reasonable and quantitative interpretation for the increase in *R*_0_. We replace the original *R*_0_ with our time-varying adjusted $R_{0}^{\prime }\left(t\right)$ in the modified model, and our study suggests that the increase in $R_{0}^{\prime }\left(t\right)$ is due to the strengthening of seasonality or to the weakening of control measures. The former is a gradual and periodic process, while the latter is a policy-decided and abrupt process, and a superposition of these two factors will lead to a large-scale outbreak.

In previous studies, a quarantine rate is often used to separate the quarantine population from the infectious population and to formulate the influences of control measures [34]. However, the quarantine rate is usually assigned manually and discretely [35]. A study on the Ebola epidemic formulated the influences of human intervention by an exponential function, but was only suitable for a single-stage epidemic [36]. Our model succeeds in establishing the continuous variation functions of the quarantine index during each period, and we multiply the periodic $R_{0}\left(t\right)$ by *q*(t) and keep the rest of the model unchanged instead of adding an additional subpopulation to the SEIR model. In the simulation, the fitted values of the parameters can vary widely and form different combinations but achieve similarly high *R*^2^ values, so all further interpretations for these parameters should be taken with caution. For example, $q_{o}$ is 0.17 in both the United Kingdom and the United States, but this does not mean that the control measures of these two countries are at the same level.

Many previous studies have indicated that the asymptomatic group and the undetected group play an important role in the transmission of the COVID-19 epidemic [37,38]. For simplicity, these two groups are not included in our model, but satisfactory adjustment performance can still be achieved. Moreover, new variants of COVID-19 (i.e., Beta; Delta; Omicron) are spreading throughout the world and dramatically changing the epidemic evolution patterns [39,40,41], and more data should be collected to verify the seasonality and intensity of their transmission ability. So far, the basic reproductive number of the newest variant Omicron has not been clearly elucidated, and it should be treated separately from other strains in the model simulation. Vaccination can effectively prevent transmission by reducing the suspected population. However, due to production limitation and validity of vaccination in new variants, herd immunity is difficult to achieve and control measures are still needed. In this paper, the influences of new variants and vaccination are excluded by shortening the study period before March 2021. If the study period is extended, our model does not perform well mainly because of these two factors.

It has been more than two years since COVID-19 appeared and swept across the world. Since there is no indication that the epidemic will disappear in a short period of time, it is of great importance to uncover the driving factors of the multistage COVID-19 epidemic, which will help policy makers to control the epidemic and avoid a large-scale outbreak. Some countries scaled down control measures only when daily confirmed cases increased to unacceptable levels, resulting in a significant number of deaths. Therefore, in terms of public health, we propose real-time calculation and monitoring of the $R_{0}^{\prime }\left(t\right)$ proposed in this paper. Once $R_{0}^{\prime }\left(t\right)$ breaks through an upward value and remains at a fairly high level for several days, control measures should be restricted in advance regardless of the strengthening of seasonality or the weakening of control measures to avoid a superposition of these two factors, since we cannot change the fluctuation of seasonality. 

## 5. Conclusions

The aim of this study is to interpret how seasonality and control measures jointly determine the multistage waves of the COVID-19 epidemic. We formulate and integrate these two factors into our modified SEIR model and test our model with data from twelve countries in two hemispheres. Our model achieves high fitness and captures the multistage patterns well, and the influences of seasonality and control measures can be well formulated and interpreted. The multilevel epidemic is shaped by the joint influence of control measures and seasonality. If only seasonality plays a role, the basic reproductive number *R*_0_ cannot fall below one and control measures must be taken. An overlay of strong seasonality and a low level of control measures can lead to a dramatically rapid increase in reported cases.

Our study also has some limitations. First, some anti-seasonal outbreaks cannot be captured by our model. Second, the use of the quarantine index relies on the exact time of the measures, and does not work well if these time points cannot be collected. Finally, other long-term factors such as new variants, vaccination, reinfection and demographics are not accounted for in our study.

## Figures and Tables

**Table 1:** Start dates of outbreak and containment periods for the selected countries in this study.

Countries	Outbreak Period 1	Containment Period 1	Outbreak Period 2	Containment Period 2	Outbreak Period 3	Containment Period 3
United States	21 March 2020	1 May 2020	NA	NA	7 February 2021	NA
France	16 March 2020	11 May 2020	3 October 2020	11 November 2020	3 April 2020	NA
Brazil	25 March 2020	1 June 2020	24 November 2020	NA	NA	NA
United Kingdom	23 March 2020	11 May 2020	5 November 2020	2 December 2020	4 January 2021	17 May 2021
Italy	9 March 2020	4 May 2020	25 October 2020	10 January 2021	3 April 2021	2 June 2021
Germany	16 March 2020	20 April 2020	12 December 2020	NA	NA	NA
Turkey	1 April 2020	12 May 2020	8 November 2020	25 January 2021	29 April 2021	17 May 2021
Australia	2 March 2020	27 April 2020	2 August 2020	13 September 2020	1 January 2021	29 January 2021
Spain	13 March 2020	13 April 2020	25 October 2020	23 November 2020	8 January 2021	9 May 2021
Argentina	20 March 2020	16 May 2020	1 July 2020	18 July 2020	26 October 2020	1 December 2020
South-Africa	26 March 2020	1 June 2020	12 July 2020	17 August 2020	29 December 2020	NA
Chile	18 March 2020	7 August 2020	3 January 2021	23 March 2021	NA	NA

**Table 2:** Upper and lower bounds of fitted parameters for the model.

Parameters	Upper and Lower Bounds
$q_{o}$	(0, 0.5]
$q_{m}$	[0.1, 0.9]
ω	[1, 365]
$A_{0}$	[0.1, 0.9]
$A_{1}$	[0.8, 2.0]
$\varepsilon_{o}$	(0, 3]
$\varepsilon_{m}$	(0, 3]

**Table 3:** Results of fitted parameters for the selected countries of this study.

Countries	ω	*A*_0_	*A*_1_	Maximum Value (*A*_1_ + *A*_0_)	Minimum Value (*A*_1_ − *A*_0_)	$q_{o}$	$q_{m}$	$\varepsilon_{o}$	$\varepsilon_{m}$
United States	86	0.56	5.32	5.88	4.76	0.17	0.21	0.25	3.00
France	121	1.07	3.58	4.65	2.51	0.13	0.47	0.05	0.02
Brazil	14	0.84	2.69	3.53	1.85	0.32	0.50	0.07	0.70
United Kingdom	98	0.50	4.14	4.64	3.64	0.17	0.31	1.02	3.00
Italy	108	1.24	4.02	5.26	2.78	0.16	0.31	0.12	0.22
Germany	110	1.25	5.17	6.42	3.92	0.16	0.23	0.48	0.96
Turkey	71	1.82	2.85	4.67	1.02	0.14	0.79	0.08	0.02
Australia	274	1.10	4.88	5.99	3.78	0.09	0.23	0.47	0.11
Spain	66	0.63	3.27	3.90	2.64	0.23	0.40	0.54	0.05
Argentina	339	0.74	4.60	5.34	3.86	0.23	0.27	0.23	3.00
South-Africa	347	1.80	4.31	6.11	2.51	0.15	0.40	0.30	0.19
Chile	339	1.27	2.60	3.87	1.33	0.31	0.69	0.09	3.00

## Data Availability

Not applicable.
